# Comparing analytical strategies for balancing site-level characteristics in stepped-wedge cluster randomized trials: a simulation study

**DOI:** 10.1186/s12874-023-02027-y

**Published:** 2023-09-12

**Authors:** Clement Ma, Alina Lee, Darren Courtney, David Castle, Wei Wang

**Affiliations:** 1 Biostatistics Core, Centre for Addiction and Mental Health, Toronto, ON Canada; 2 Center for Complex Interventions, Centre for Addiction and Mental Health, Toronto, ON Canada; 3 Dalla Lana School of Public Health, University of Toronto, Toronto, ON Canada; 4 Cundill Centre for Child and Youth Depression, Centre for Addiction and Mental Health, Toronto, ON Canada; 5 Department of Psychiatry, University of Toronto, Toronto, ON Canada; 6 Department of Psychiatry, University of Tasmania, Hobart, TAS Australia; 7 Centre for Mental Health Service Innovation, Statewide Mental Health Service, Hobart, TAS Australia; 8 College of Public Health, University of South Florida, Tampa, FL USA

**Keywords:** Stepped-wedge cluster randomized trials, Cluster-level imbalance, Simulation

## Abstract

**Background:**

Stepped-wedge cluster randomized trials (SWCRTs) are a type of cluster-randomized trial in which clusters are randomized to cross-over to the active intervention sequentially at regular intervals during the study period. For SWCRTs, sequential imbalances of cluster-level characteristics across the random sequence of clusters may lead to biased estimation. Our study aims to examine the effects of balancing cluster-level characteristics in SWCRTs.

**Methods:**

To quantify the level of cluster-level imbalance, a novel imbalance index was developed based on the Spearman correlation and rank regression of the cluster-level characteristic with the cross-over timepoints. A simulation study was conducted to assess the impact of sequential cluster-level imbalances across different scenarios varying the: number of sites (clusters), sample size, number of cross-over timepoints, site-level intra-cluster correlation coefficient (ICC), and effect sizes. SWCRTs assumed either an immediate “constant” treatment effect, or a gradual “learning” treatment effect which increases over time after crossing over to the active intervention. Key performance metrics included the relative root mean square error (RRMSE) and relative mean bias.

**Results:**

Fully-balanced designs almost always had the highest efficiency, as measured by the RRMSE, regardless of the number of sites, ICC, effect size, or sample sizes at each time for SWCRTs with learning effect. A consistent decreasing trend of efficiency was observed by increasing RRMSE as imbalance increased. For example, for a 12-site study with 20 participants per site/timepoint and ICC of 0.10, between the most balanced and least balanced designs, the RRMSE efficiency loss ranged from 52.5% to 191.9%. In addition, the RRMSE was decreased for larger sample sizes, larger number of sites, smaller ICC, and larger effect sizes. The impact of pre-balancing diminished when there was no learning effect.

**Conclusion:**

The impact of pre-balancing on preventing efficiency loss was easily observed when there was a learning effect. This suggests benefit of pre-balancing with respect to impacting factors of treatment effects.

**Supplementary Information:**

The online version contains supplementary material available at 10.1186/s12874-023-02027-y.

## Background

**Fig. 1:**
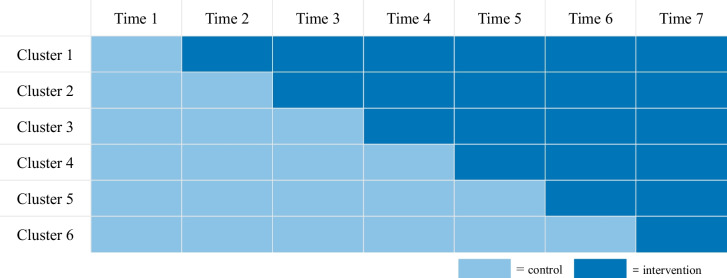
A hypothetical SWCRT with 6 clusters and 7 time points

Stepped-wedge cluster randomized trials (SWCRTs) are a relatively novel type of cluster-randomized trials (CRTs) in which the active intervention is implemented at cluster level, e.g. hospitals, clinics, schools and etc., with participants nested within each cluster [1]. All clusters start with the control intervention, and then clusters are randomized to cross-over to the active intervention sequentially at regular intervals during the study period (Fig. 1). SWCRTs have been implemented in a wide number of research areas, including: human immunodeficiency virus (HIV), cancer, healthcare associated infections, social policy, and criminal justice. This design can be appealing to test interventions that can only be delivered at a cluster level. Further, SWCRTs may be attractive to participating sites and implementing groups as all study sites will cross-over to the active intervention [1]. SWCRTs also increase power relative to a traditional parallel CRTs in situations where a high intra-cluster correlation is anticipated [1].

For randomized trials, imbalances in baseline characteristics between treatment arms may occur [2, 3]. For trials randomized at the participant level, a number of strategies to reduce the chance of covariate imbalance have been proposed, including: stratified randomization [4], minimization [5], covariate-adaptive randomization [6], minimal sufficient balance randomization [7]. Analytical strategies to account for covariate imbalance include: adjusting for pre-specified confounders. For cluster randomized trials, given the usually small number of clusters, there is an increased chance of covariate imbalance between clusters randomized to the treatment and control interventions [8]. Stratification, minimization, “best balance” allocation [9], and covariate-constrained randomization [10] can similarly reduce the chance for covariate imbalance in CRTs. Direct adjustment of covariates in linear mixed models can reduce estimation bias and prevent power loss compared to unadjusted models [11].

For SWCRTs, sequential imbalances across the random sequence of clusters may be an issue; e.g. cluster-level characteristics systematically differed between clusters randomized early to crossover to the intervention treatment versus clusters randomized later in the trial. For example, consider a 6-site SWCRT where clinicians at each site have differing average years of experience that can be categorized to low [L], moderate [M], or high [H]. If randomization was left to chance, the sequence of sites may be {L, L, M, M, H, H}. Thus, this introduces a linear/sequential imbalance across the 6 sites with sites with high levels of training being under-represented in the study due to later entrance. While this is a hypothetical extreme case of low probability, other sequences of less severe sequential imbalance may occur more frequently, especially given that SWCRTs usually randomize a small number of clusters [12]. In addition, site-level covariates may be imbalanced in a non-linear (e.g. quadratic) or cyclical (e.g. seasonal) manner.

As a motivating example, the Canadian Institutes of Health Research (CIHR) funded project grant, titled *Effectiveness of an Integrated Care Pathway for Adolescent Depression: a Multi-site Stepped-Wedge, Cluster-Randomized Controlled Trial (CARIBOU-2)*, aims to implement a complex intervention with six sites sequentially using the SWCRTs design [13]. The CARIBOU-2 study aims to test the effectiveness and implementation of an Integrated Care Pathway (ICP) (i.e., a multidisciplinary treatment algorithm) for the treatment of depression in adolescents based on the highest quality practice guidelines. The ICP is intended to facilitate the delivery of coordinated evidence-based treatments at the clinic level. Depression is the leading cause of disability in adolescents and a potent risk factor for adolescent suicide. Evidence-based treatments are available; however many clinics do not provide guidelines-based treatments. Whether or not the ICP actually leads to improved outcomes for depression in adolescents is still unknown and testing this approach is a complex undertaking. This project aims to use an SWCRT design where adolescents with depression are allocated to the ICP versus treatment-as-usual (TAU) in community settings. Participants will consist of adolescents (age 13 to 18) with depressive symptoms presenting to one of six community mental health agencies across the province of Ontario, which will be admitted in the stepped-wedge design in seven timepoints. The primary outcome is change in depressive symptoms from baseline to the 6-month time point. Potential site-level factors that may impact treatment outcome include rurality, income-level of the neighborhood communities, and average years of experience of the clinicians. Given the age group of the participants, both linear and seasonal site-level imbalances may be observed.

Stratification and covariate-constrained randomization can help reduce the effects of covariate imbalance in SWCRTs [14]. Lew et al. (2019) [15] proposed a metric to quantify sequential site-level imbalances. Sequential balancing of covariates was achieved by calculating the imbalance metric for site-level characteristics for all possible site assignments and selecting the randomization sequence that minimized the imbalance metric. There lacks a unified approach to quantify different, multiple types of imbalances, including linear, non-linear, and cyclical imbalances. Analysis of SWCRTs typically use linear mixed effects models to estimate treatment effects [16]; analysis using generalized estimating equations or non-parametric methods have also been proposed [17]. Prior studies have examined the impact of unequal cluster sizes on sample size and power [18] in SWCRTs [19, 20]. However, the field still lacks research studies that quantifies the association between magnitude of imbalance and potential bias and efficiency in assessing impact of the active intervention.

In this study, we examine the impact on estimation bias and efficiency of not prospectively balancing as a function of magnitude of imbalance, as well as design features as covariates. In particular, we focus on a model that incorporates a cumulative treatment effect over time [16]. This can be observed when the training of the interventionist is required and thus the treatment effect may increase over time, starting from the time of cross-over to the active treatment until saturation. This is referred to as the “learning effect” in the paper in contrast to a constant (or immediate) treatment effect. We further assumed that the treatment effect did not depend on the time it was initiated [1, 21]. We also introduce a standardized definition of imbalance index and propose methods of incorporating multiple imbalance metrics and multiple site-level factors. We conducted a simulation study to demonstrate the benefit of balancing site-level factors across a wide spectrum of scenarios by varying the: number of sites, sample size per site, number of steps of the design, and effect sizes.

## Methods

This paper introduces methods to balance SWCRTs at the randomization stage in order to cope with linear, non-linear and seasonal effects in terms of time of transition from control/waiting to active intervention condition. We examine the impact of not proactively balancing, methods of balancing including incorporating multiple temporal factors and multiple site-level factors. A simulation study focused on balancing linear impact has been conducted to demonstrate the benefit of balancing in a wide spectrum of scenarios taking into account factors of number of sites, sample size at each site, number of steps of the design, and perceived magnitude of effect sizes. Extensive sensitivity analyses examined alternative models which include the random effect on the treatment effect at the site level, and at the site by time level. The balancing based randomization will also be demonstrated on the motivating study.

### Linear, non-linear and seasonal site-level imbalances

We discuss three types of effects that may impact the evaluation of the treatment efficacy: (1) linear/sequential, (2) non-linear, and (3) seasonal imbalance across site-level characteristics. For example in the CARIBOU-2 study, the rurality varies across the six sites and each site can be classified as urban, suburban, or rural, and subsequently coded as 0, 1, and 2 depending on level of rurality. Without considering the balance of site-level characteristics, randomization may generate the following sequence of six sites with linear/sequential imbalance: {0, 0, 1, 1, 2, 2}. Intuitively, sites with high rurality will be severely under-represented in the novel ICP intervention condition due to later entrance.

To quantify the linear or sequential imbalance, we define imblancedness index, ${i}_{L}$ as the absolute value of the Spearman’s correlation coefficient between quantified site characteristics and time, $\{t, t=1, 2,.., T\}$. It ranges from 0 (perfectly balanced) to 1 (perfectly imbalanced). Equivalently, it can also be defined as the square root of the coefficient of determination, commonly known as the R-squared, of the regression of which the ranked site characteristics is regressed on the sequential time indices.

The same approach can be extended to evaluate imbalance for specific non-linear patterns. For example, we define the quadratic imbalance index, ${i}_{Q}$, as the absolute value of the partial Spearman correlation coefficient between the quantified site characteristics and squared time, $\{{t}^{2}, t=1, 2,.., T\}$, with linear time being controlled. It ranges from 0 (perfectly balanced) to 1 (perfectly imbalanced) as well. Or in regression terms, this is the square root of the partial coefficient of determination of the regression of which the ranked site characteristics is regressed on the quadratic time with the sequential time controlled in the model.

Similarly, we define the seasonal imbalance index, ${i}_{s}$, as the square root of the coefficient of partial determination when the ranked site characteristics is regressed on seasonal indicators with sequential time trend and/or other time trend, e.g. quadratic, controlled.

**Fig. 2:**
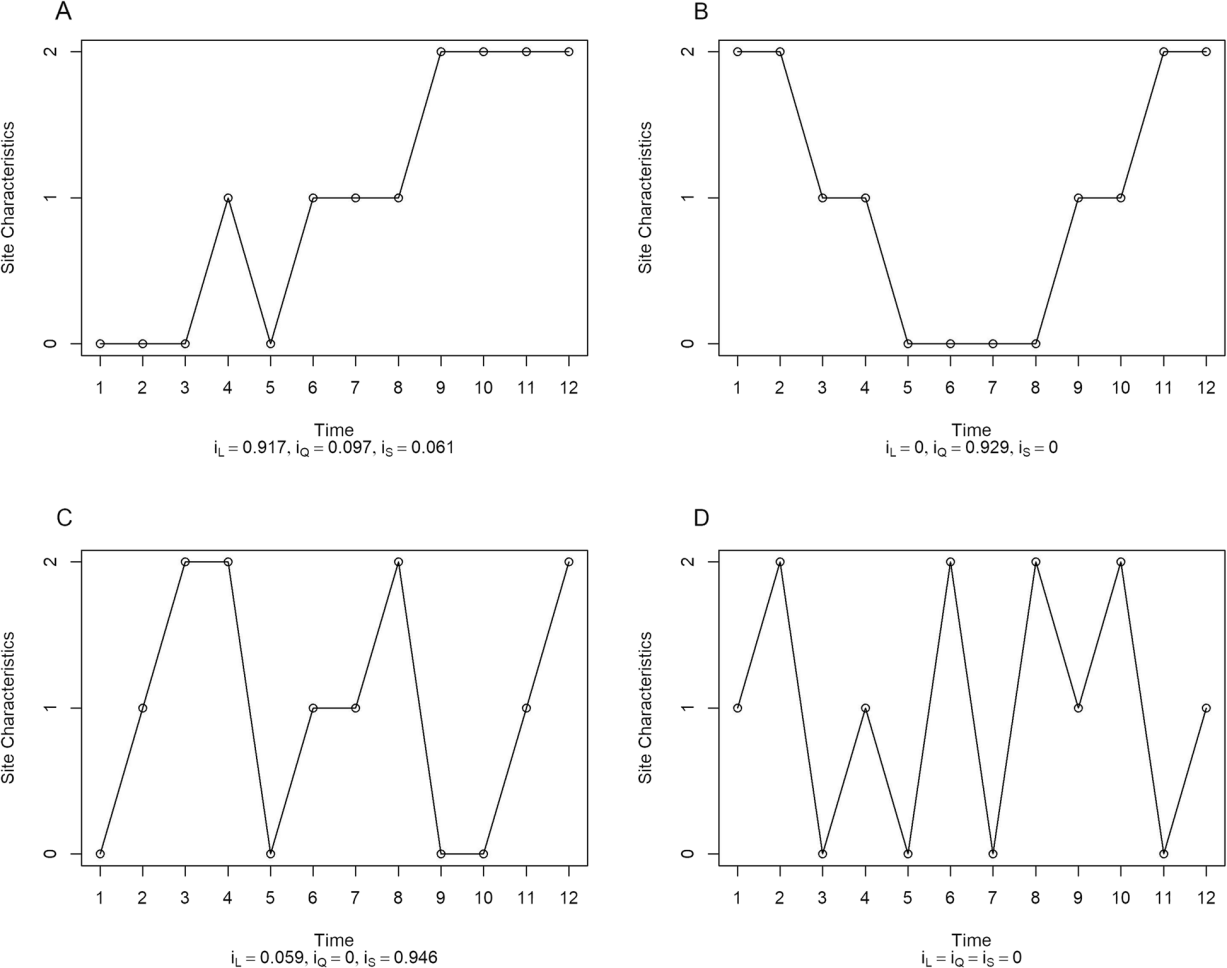
Example diagrams of imbalance at linear, quadratic, and seasonal levels

Figure 2 shows four hypothetical randomization sequences with varying degrees of linear, quadratic and seasonal imbalance assuming a SWCRT with 12 sites and one ordinal site-level characteristic with three levels, denoted as 0, 1 and 2. The y-axis represents the level of the site characteristic and x-axis represents the time of each site crossing over from control to active treatment. Every four steps constitutes a full yearly cycle for seasonal effects. The first sequence (Fig. 2A) is linearly imbalanced (*i*_*L*_ = 0.917) with minimal quadratic and seasonal imbalance. The subsequent sequences show quadratic imbalance (Fig. 2B), seasonal imbalance (Fig. 2C), and fully balanced (Fig. 2D) randomization sequences. For example, as Fig. 2C demonstrated that sites of Level 0 all appeared earlier in the seasons, Level 1 sites, more in the midseason, and Level 2 sites, near the end of the seasons. This showed a strong cyclic or seasonal trend (${i}_{S}=0.946$) and it may impact the estimation when there is an interaction between seasons and treatment effect.

When there are multiple site characteristics and/or multiple linear or non-linear trends to be balanced, we define the overall imbalance index, *i*_*o*_, as a weighted average of individual imbalance indices, *i*_*k*_, as defined in the previous section:

1 $$i_o=\Sigma{w_ki}_k,\mathrm{where}\;\Sigma w_k=1$$

The weights, $\left\{{w}_{k}, k=1, 2, \dots \right\}$, should be determined by the content experts of the trial.

### Strategy of balancing

To ensure balance of site-level characteristics in the SWCRT, we recommend the following strategy to minimize the overall imbalance of the design.

#### Step 1: Determine the site characteristics to balance

These are the moderators of the treatment effects at the site level. The knowledge could be obtained from literature review, pilot data, or a panel of content experts in the field. Examples include: size of the clinics/sites, income levels of the communities of the study sample, rurality (rural, suburban, urban, or mixer of any of the above), and others. If multiple site-level characteristics are selected, it is recommended to check for multi-collinearity by calculating the pairwise correlation between characteristics.

#### Step 2: Determine the time trend(s) to balance

Most commonly, it is sufficient to only balance the linear trend. The quadratic trend may be needed if non-linear associations between the site-level characteristic and the treatment effect are expected. The seasonal trend may be of interest, for example, when the population consists of school aged children or youth as their performance may be impacted by school semesters and yearlong schedule [22].

#### Step 3: If multiple indices need to balanced, determine the weight of each index

The weights can be determined by a panel of experts. One approach is to use a pre-determined ranking system or rating system that evaluates the importance of the type of trend and site characteristics, especially when directly determining numerical weights is difficult. The ranks or ratings then can be converted to weights [23, 24]. For example, if there are *K* indices to be balanced and they are ranked from the most important ($$r=1)$$ to the least important ($$r=K),$$ an index or rank *r* can be assigned with a weight of $${\left(K-r+1\right)}^{p}$$*,* of which the exponent *p* is a parameter to control the distribution of the weights. Please also note while all the indices have the same range of 0 to 1, the performance of their combination can be complicated and may require intricate statistical knowledge as well as domain knowledge.

#### Step 4: Calculate the overall imbalance across randomization sequences and randomly select a sequence that minimizes imbalance

For a small number of study sites, exhaustive enumeration of all randomization sequences may be feasible. For a larger number of sites, a large but not exhaustive set of randomization sequences can be randomly generated. Calculate the overall imbalance for each sequence and randomly select a sequence that minimizes imbalance.

Overly covariate-constrained randomizations could jeopardize the impartiality of the study design [25]. For example, to achieve the minimum of the imbalance index, some sites may have to be assigned in a specific sequence. We may need to relax the minimization requirement if only a few design options are available for selection.

### Distribution of imbalance index

To assess the distribution of imbalance index, we exhaustively enumerated all permutations of the 6-site and 12-site settings and computed the linear imbalance index of these designs. We assume the cluster-level characteristics have three ordinal levels noted by 0, 1 and 2 with 2 or 4 sites at each level. There are 90 and 34,650 unique designs in terms of the sequential distribution of the site characteristics for 6-site and 12-site settings, respectively. Descriptive statistics were used to summarize the distribution of imbalance indices for the two settings.

### Simulation study design

We performed a simulation study to evaluate how sequential\linear imbalance may impact the precision of estimating the treatment effect in stepped-wedge cluster randomized trials. The primary and secondary evaluation criteria are the relative root mean square error (RRMSE) and relative mean bias of the estimate, respectively. They are used to compare the population average effects, preset by the simulation model, and the sample average treatment effect, obtained by simulated data sets, in terms of efficiency and unbiasedness, respectively.

Each simulated trial assumed $i = 1,\dots , I$ clusters, $t = 1,\dots , T$ timepoints, and $j = 1,\dots , J$ participants per cluster per time point. The SWCRT assumed a cross-sectional, rather than a longitudinal design, to mirror the design of the CARIBOU study. The total sample size was $N = I \times J \times T$. The continuous outcome for the *j*^*th*^ participant in cluster *i* at time *t*, was denoted as ${Y}_{ijt}$. The cluster-level characteristics for the *i*^*th*^ cluster were denoted as ${Z}_{i}$. The treatment indicator for the *i*^*th*^ cluster at time *t* was ${Group}_{i,t}$ with ${Group}_{i,t}=1$ for the active intervention and ${Group}_{i,t}=0$ for the control intervention.

The SWCRT design assumed either: (1) a constant treatment effect; or (2) an increasing treatment effect over time (e.g. a learning effect).

The primary SWCRT model with constant treatment effect is given by Eq. (2):

2 $${Y}_{ijt}={\beta }_{0}{+{b}_{0i}+\beta }_{1}*{Z}_{i}*{Group}_{i,t}{{+{\varvec{\beta}}}_{2}*{\varvec{\tau}}+\epsilon }_{ijt}$$

$$where\;{Group}_{i,t}=\left\{\begin{array}{c}0,t<T_i\\1,t\geq T_i\end{array}\right.and$$

$$T_i=Time\;where\;i^{th}cluster\;is\;assigned\;to\;active\;intervention$$

The treatment effect ${\beta }_{1}$ remains constant across all timepoints after the *i*^*th*^ cluster crosses-over to the active intervention at time *T*_*i*_. However, the treatment effect varies between clusters depending on the cluster-level characteristics *Z*_*i*_. The random error is ${\epsilon }_{ijt}\sim N(0,{\sigma }^{2})$. The clustering effect is denoted by the random effect ${b}_{0i}$, which follows $N(0,{\sigma }_{re}^{2})$ and is independent of ${\epsilon }_{ijt}$. Also included in the model is a time effect denoted by ${{\varvec{\beta}}}_{2}*{\varvec{\tau}}$, of which ${\varvec{\tau}}$ represents a categorical time indicator with $T+1$ levels. For all the simulations, we have set ${{\varvec{\beta}}}_{2}=0$ but included the term in the estimation model to better reflect the practice of SWCRT in the field.

The primary SWCRT model with a linear learning effect is given by Eq. (3):

3 $${Y}_{ijt}={\beta }_{0}{+{b}_{0i}+\beta }_{1}*{f}_{i}\left(t\right)*{Z}_{i}*{Group}_{i,t}{{+{\varvec{\beta}}}_{2}*{\varvec{\tau}}+\epsilon }_{ijt}$$

$$\mathrm{where}\;{Group}_{i,t}=\left\{\begin{array}{c}0,t<T_i\\1,t\geq T_i\end{array}\right.,$$

$${f}_{i}\left(t\right)=\left\{\begin{array}{c}0, t <{T}_{i}\\ (t - {T}_{i}+1)/(T-1), t \ge {T}_{i}\end{array}\right. and$$

$$T_i=\mathrm{Time}\;\mathrm{where}\;\mathrm{ith}\;\mathrm{cluster}\;\mathrm{is}\;\mathrm{assigned}\;\mathrm{to}\;\mathrm{active}\;\mathrm{intervention}$$

The function ${f}_{i}\left(t\right)$ specifies the learning effect of cluster *i* at time *t*. The effect size increases by increments of ${\beta }_{1}$ at each successive time point after the *i*^*th*^ cluster has crossed-over to the active intervention at time *T*_*i*_. Thus, the overall treatment effect is ${\beta }_{1}*{f}_{i}\left(t\right)$. For example, the first cluster to cross over will have effective effect sizes of ${\beta }_{1}/(T-1)$ and ${\beta }_{1}$ at time $t=2$ and $t=T$, respectively, with increment of ${\beta }_{1}/(T-1)$ at each step after crossover and only the first site achieved the full effect size at the final step.

As a sensitivity analysis, we examined two variations to the primary SWCRT models. Assuming constant treatment effects, the first variation which includes an additional random effect at the cluster level, ${b}_{1i}$, to the treatment coefficient ${\beta }_{1}$ is given by Eq. (4).

4 $${Y}_{ijt}={\beta }_{0}{+{b}_{0i}+(\beta }_{1}+{b}_{1i})*{Z}_{i}*{Group}_{i,t}{{+{\varvec{\beta}}}_{2}*{\varvec{\tau}}+\epsilon }_{ijt}$$

Assuming constant treatment effects, the second variation is augmented by an additional random effect at the cluster by time level, denoted by ${b}_{0i,t}$ [Eq. (5)].

5 $${Y}_{ijt}={\beta }_{0}{+{b}_{0i}+{b}_{0i,t}+\beta }_{1}*{Z}_{i}*{Group}_{i,t}{{+{\varvec{\beta}}}_{2}*{\varvec{\tau}}+\epsilon }_{ijt}$$

These variations have also been similarly applied to the simulations for the learning effects models.

### Simulation parameters

Simulations were performed using *R* version 4.1.3*.* Trials assumed $I =6$ clusters and $T=7$ steps, or $I = 12$ clusters and $T = 13$ steps. Trials had $J = 10$ or 20 participants per cluster at each step. The $I+1$ steps allows all clusters to begin on the control intervention at $t = 1$.

For the primary models with constant effect, the treatment effect was ${\beta }_{1}$ = 0.2, 0.5, or 1.0 for all trials. For the model with a learning effect, the incremental effect sizes were determined as ${\beta }_{1}/(T-1)$. Thus, the incremental effect sizes were 0.033, 0.083, or 0.0167 for the 6-site designs, and 0.017, 0.042, and 0.083 for the 12-site designs. These incremental effect sizes achieved the same full effect size (${\beta }_{1}$ = 0.2, 0.5, or 1.0) at the final step for the first site to crossover. Throughout the simulation, the standard deviation of the random error, $\sigma$, was assumed to be 1. The clustering effects was controlled by the ICC, $\frac{{\sigma }_{re}^{2}}{{\sigma }_{re}^{2}+{\sigma }^{2}}$. Two levels of ICC for the control condition, 0.01 and 0.10, were used in the simulation.

The cluster-level characteristics were ordinal with three levels (e.g. 0 = small, 1 = medium, 2 = large) with even number of sites distributed on each level. Site level characteristics were coded as $Z= \{\mathrm{0,0},\mathrm{1,1},\mathrm{2,2}\}$ or $Z= \{\mathrm{0,0},\mathrm{0,0},\mathrm{1,1},\mathrm{1,1},\mathrm{2,2},\mathrm{2,2}\}$ for trials with 6 or 12 clusters, respectively. To demonstrate the impact of different level of imbalance, we selected sequences of $Z$ that have imbalance indices at 0^th^, 33^th^, 66^th^, 83^th^, and 100^th^ percentiles of all permuted sequences (90 for 6 sites and 34,650 for 12 sites). For.

In total, we have examined 240 scenarios varying six parameters: number of sites (6 and 12), sample size per step/site (10 and 20), type of learning effects (constant and increasing/learning), effect sizes (0.2, 0.5, and 1.0), ICC (0.01 and 0.10), and level of imbalance (0^th^, 33^th^, 66^th^, 83^th^, and 100^th^ percentiles of the permuted distribution). Each scenario has been simulated 10,000 times. As a sensitivity analysis, specific scenarios were simulated 50,000 times to confirm the reliability of estimates. Linear mixed-effects modeling accounting for site-level clustering using site-level random intercept and random effect for ${\beta }_{1}$ were used to estimate the sample average treatment effects. For example, for the scenario with constant treatment effect, the estimates were obtained by the following model,

6 $${Y}_{ijt}={\beta }_{0}{+{b}_{0i}+(\beta }_{1}+{b}_{i1})*{Group}_{i,t}{{+{\varvec{\beta}}}_{2}*{\varvec{\tau}}+\epsilon }_{ijt}$$

While Eq. (6) differs from Eq. (2), it does not require the knowledge of clustering level characteristics (or potential misclassification) but still obtains an unbiased estimate of ${\beta }_{1}$ by incorporating the random effect ${b}_{i1}$.

All the aforementioned simulations metrics have also been applied the secondary models. For Model (4), the standard deviation of ${b}_{i1}$ was set to be 10% of the corresponding ${\beta }_{1}$ value. For Model (5), the standard deviation of ${b}_{0i,t}$ was fixed at 0.10. For this model, the fitted model also included a cluster by time random effect.

### Simulation metrics

Relative root mean square error (RRMSE) and relative mean bias using the following formulae were employed to evaluate the performance of different designs by comparing estimated sample average treatment effects with the true treatment effects specified by the simulation design (${\beta }_{1}$).

Relative root mean square error (RRMSE)

$$RRMSE\left({\widehat{\beta }}_{1}\right)=\sqrt{\frac{1}{K}{\sum }_{k=1}^{K}{({\widehat{\beta }}_{1,k}-{\beta }_{1})}^{2}}/{\beta }_{1}$$

Relative mean bias

$$Bias\left({\widehat{\beta }}_{1}\right)=\frac{1}{K}\left|{\sum }_{k=1}^{K}\left({\widehat{\beta }}_{1,k}-{\beta }_{1}\right)\right|/{\beta }_{1}$$

where ${\widehat{\beta }}_{1,k}$ is the estimate from the *k*-th simulation (*k* = 1, …, *K* = 10,000).

## Results

### Distribution of imbalance index in 6- and 12-site SWCRTs

Using exhaustive enumeration, we assessed the distribution of imbalance indices for 6- and 12-site SWCRTs (Table 1). For the 6-site SWCRTs, the median imbalance index was 0.359 (range 0 to 0.956). The first and second tertiles were 0.239 and 0.478, respectively. One sixth of the designs have an imbalance index of 0.717 or higher. For the 12-site design, the median imbalance index was 0.207 (range 0 to 0.946). The first and second tertiles were 0.148 and 0.296 respectively. One sixth of the designs have an imbalance score of 0.414 or higher. With a larger number of sites, the mean of the distribution of the imbalance index shifts towards zero.

**Table 1:** Distribution of imbalance indices in 6- and 12-site SWCRTs

			X^th^ Percentile						
# Sites	# Timepoints	# Unique designs	0^th^	16.7^th^	33^rd^	50^th^	67^th^	83^rd^	100^th^
6	7	90	0	0.119	0.239	0.359	0.478	0.717	0.956
12	13	34,650	0	0.059	0.148	0.207	0.296	0.414	0.946

### Simulation results for SWCRT with learning effect

The impact of pre-balancing can be easily observed when there is a hypothesized learning effect: it is easy to conclude that pre-balanced design will always have the highest efficiency (measured by RRMSE) regardless of the ICC, the number of sites or sample size at each site/time. At ICC = 0.01, for SWCRTs with 6 sites and 10 participants recruited at each step and each site, we saw a consistent decreasing trend of efficiency, i.e. increasing RRMSE’s as imbalance increased (Table 2). For example, for an overall effect size of 0.5, the RRMSE decreased from 1.225 for fully imbalanced (100^th^ percentile) designs to 0.738 for fully balanced designs (0^th^ percentile). This represents a maximal efficiency loss of 66.0% (e.g. [1.225–0.738]/0.738). Even switching from a fully-balanced design to a partially imbalanced design (33^rd^ percentile) will have an efficiency loss of 61.6% (e.g. [1.225–0.758]/0.758). This trend repeats at ICC = 0.10.

**Table 2:** Relative Root Mean Square Error (RRMSE) of the estimated treatment effects

ICC	Number of sites	Sample size at each step	Imbalance index	Percentiles	With learning effect			With constant effect		
					Effect size			Effect size		
					0.2	0.5	1.0	0.2	0.5	1.0
0.01^a^	6	10	0.000	0^th^	1.646	0.738	0.444	0.747	0.304	0.158
			0.239	33^rd^	1.653	0.758	0.472	0.748	0.314	0.159
			0.478	67^th^	1.733	0.848	0.540	0.782	0.327	0.163
			0.717	83^rd^	1.824	1.015	0.699	0.799	0.341	0.167
			0.956	100^th^	1.951	1.225	0.964	0.851	0.373	0.18
		20	0.000	0^th^	1.295	0.610	0.376	0.557	0.227	0.115
			0.239	33^rd^	1.322	0.632	0.396	0.56	0.229	0.114
			0.478	67^th^	1.383	0.717	0.453	0.568	0.236	0.117
			0.717	83^rd^	1.515	0.870	0.569	0.589	0.242	0.121
			0.956	100^th^	1.630	1.121	0.813	0.627	0.253	0.123
	12	10	0.000	0^th^	0.896	0.409	0.253	0.389	0.161	0.082
			0.148	33^rd^	0.902	0.430	0.264	0.395	0.162	0.083
			0.296	67^th^	0.956	0.488	0.310	0.396	0.164	0.083
			0.414	83^rd^	0.993	0.542	0.361	0.414	0.168	0.085
			0.946	100^th^	1.365	1.060	0.835	0.491	0.197	0.094
		20	0.000	0^th^	0.711	0.336	0.215	0.291	0.118	0.060
			0.148	33^rd^	0.721	0.348	0.227	0.292	0.119	0.060
			0.296	67^th^	0.780	0.399	0.262	0.297	0.120	0.060
			0.414	83^rd^	0.812	0.452	0.298	0.296	0.120	0.061
			0.946	100^th^	1.232	0.968	0.669	0.339	0.131	0.064
0.10^b^	6	10	0.000	0^th^	2.238	0.955	0.548	0.855	0.341	0.173
			0.239	33^rd^	2.223	0.968	0.569	0.849	0.348	0.173
			0.478	67^th^	2.314	1.042	0.632	0.860	0.351	0.173
			0.717	83^rd^	2.349	1.169	0.775	0.849	0.349	0.173
			0.956	100^th^	2.453	1.324	0.997	0.859	0.356	0.179
		20	0.000	0^th^	1.656	0.752	0.437	0.614	0.249	0.124
			0.239	33^rd^	1.706	0.768	0.460	0.615	0.249	0.124
			0.478	67^th^	1.736	0.819	0.513	0.610	0.249	0.124
			0.717	83^rd^	1.823	0.964	0.621	0.606	0.248	0.126
			0.956	100^th^	1.930	1.159	0.841	0.614	0.248	0.123
	12	10	0.000	0^th^	1.134	0.497	0.292	0.440	0.178	0.089
			0.148	33^rd^	1.145	0.515	0.300	0.443	0.178	0.089
			0.296	67^th^	1.188	0.562	0.346	0.437	0.178	0.089
			0.414	83^rd^	1.209	0.610	0.395	0.449	0.178	0.090
			0.946	100^th^	1.505	1.072	0.852	0.440	0.183	0.093
		20	0.000	0^th^	0.840	0.381	0.233	0.318	0.127	0.064
			0.148	33^rd^	0.845	0.392	0.245	0.318	0.128	0.064
			0.296	67^th^	0.892	0.436	0.280	0.318	0.128	0.064
			0.414	83^rd^	0.926	0.490	0.316	0.317	0.127	0.064
			0.946	100^th^	1.281	0.974	0.680	0.313	0.127	0.064

^a^The ICC for the treatment condition for the 6-site SWCRTs were 0.040, 0.174, and 0.448 for the three effect sizes, 0.20, 0.50, and 1.00, respectively. The ICC for the treatment condition in the 12-site SWCRTs were 0.038, 0.161, and 0.424 for the three effect sizes respectively

^b^The ICC for the treatment conditions for the 6-site SWCRT were 0.125, 0.237, and 0.477 for the three effect sizes respectively. The ICC for the treatment conditions for the 12-site SWCRTs were 0.122, 0.226, and 0.456 for the three effect sizes respectively

As expected, as the ICC increases, the RRMSE increases in general. For example, the RRMSE for a partially imbalanced design (33^rd^ percentile) and effect size of 0.5 increases from 0.758 to 0.968 when the ICC increases from 0.01 to 0.10. These findings replicated for the designs at both ICC levels with 6 sites and 20 individual recruited at each stage/site with the same steady decreasing trend of efficiency over the increasing imbalance indices. Switching from the fully balanced design to the fully imbalanced design showed a maximal loss in efficiency ranging from 16.5% (effect size = 0.2, ICC = 0.10) to 116.2% (effect size = 1.0, ICC = 0.01).

Further, for the designs with 12 sites, the efficiency losses were even more substantial. Between the most balanced and least balanced designs, the efficiency loss ranged from 52.3% to 230.0% (10 individuals per site, ICC = 0.01), 10.2% to 115.7% (10 individuals per site, ICC = 0.10), 73.3% to 211.2% (20 individuals per site, ICC = 0.01), and from 52.5% to 191.9% (20 individuals per site, ICC = 0.10). Also worth noting is that for the models not controlling for site level variation, it generates the smallest RRMSE across the board. In other words, if the site level characteristics were perfectly balanced, there is no need to control for site difference. In addition to the virtually monotonic relationship between efficiency and imbalance index, we have also observed that the RRMSE decreases in general for larger sample size, larger number of sites, smaller ICC and larger effect sizes.

In terms of unbiasedness, all designs performed well (Table 3).

**Table 3:** Relative mean bias of the estimated treatment effects

ICC	Number of sites	Sample size at each step	Imbalance index	Percentiles	With learning effect			With constant effect		
					Effect size			Effect size		
					0.2	0.5	1.0	0.2	0.5	1.0
0.01	6	10	0.000	0^th^	0.016	0.007	0.004	0.005	0.003	0.001
			0.239	33^rd^	0.011	0.004	0.001	0.004	0.001	0.001
			0.478	67^th^	0.013	0.003	0.005	0.001	0.001	0.001
			0.717	83^rd^	0.013	0.005	0.004	0.001	0.001	0.001
			0.956	100^th^	0.015	0.007	0.000	0.011	0.005	0.001
		20	0.000	0^th^	0.004	0.000	0.005	0.000	0.003	0.001
			0.239	33^rd^	0.001	0.004	0.002	0.002	0.003	0.000
			0.478	67^th^	0.001	0.006	0.009	0.000	0.002	0.002
			0.717	83^rd^	0.011	0.009	0.009	0.005	0.001	0.002
			0.956	100^th^	0.007	0.008	0.002	0.006	0.002	0.000
	12	10	0.000	0^th^	0.007	0.002	0.001	0.002	0.002	0.000
			0.148	33^rd^	0.001	0.000	0.001	0.003	0.000	0.001
			0.296	67^th^	0.006	0.008	0.003	0.001	0.001	0.001
			0.414	83^rd^	0.012	0.015	0.004	0.004	0.001	0.001
			0.946	100^th^	0.002	0.005	0.004	0.004	0.001	0.000
		20	0.000	0^th^	0.005	0.003	0.002	0.002	0.001	0.001
			0.148	33^rd^	0.011	0.002	0.002	0.001	0.002	0.001
			0.296	67^th^	0.001	0.003	0.002	0.000	0.001	0.000
			0.414	83^rd^	0.012	0.000	0.002	0.002	0.002	0.001
			0.946	100^th^	0.018	0.006	0.002	0.006	0.001	0.000
0.10	6	10	0.000	0^th^	0.010	0.010	0.001	0.007	0.003	0.000
			0.239	33^rd^	0.012	0.001	0.001	0.008	0.000	0.002
			0.478	67^th^	0.039	0.003	0.006	0.009	0.001	0.001
			0.717	83^rd^	0.019	0.007	0.001	0.004	0.002	0.001
			0.956	100^th^	0.035	0.012	0.001	0.016	0.005	0.001
		20	0.000	0^th^	0.017	0.006	0.002	0.002	0.004	0.001
			0.239	33^rd^	0.008	0.003	0.004	0.004	0.002	0.000
			0.478	67^th^	0.002	0.008	0.010	0.002	0.002	0.002
			0.717	83^rd^	0.003	0.001	0.012	0.007	0.002	0.002
			0.956	100^th^	0.007	0.003	0.003	0.007	0.001	0.000
	12	10	0.000	0^th^	0.010	0.007	0.002	0.003	0.002	0.000
			0.148	33^rd^	0.000	0.004	0.001	0.004	0.000	0.001
			0.296	67^th^	0.005	0.011	0.003	0.001	0.001	0.001
			0.414	83^rd^	0.008	0.014	0.001	0.001	0.002	0.001
			0.946	100^th^	0.002	0.007	0.005	0.004	0.001	0.000
		20	0.000	0^th^	0.006	0.004	0.001	0.002	0.001	0.001
			0.148	33^rd^	0.016	0.002	0.002	0.001	0.001	0.001
			0.296	67^th^	0.000	0.005	0.002	0.002	0.001	0.001
			0.414	83^rd^	0.012	0.001	0.001	0.002	0.002	0.001
			0.946	100^th^	0.016	0.005	0.002	0.005	0.001	0.000

### Simulation results for SWCRT with constant effect

The impact of pre-balancing diminishes when there is no learning effect, i.e., the treatment effect maximizes as soon as a site is transferred from waitlist to active treatment (Table 2). While there were some subtle trend in the estimates, nothing really has stood out if we take into account of the margin of error dictated by the number of replications. In terms of unbiasedness, all designs performed well with low mean bias overall (Table 3). In Tables 2, we also reported the ICC for the intervention conditions in the footnote. Given the hypothesized varying response to the treatment due to difference in the site characteristics, the ICC for the treatment sites is highly dependent on the treatment effect size. This differs from the conventional assumption that the varying response to the treatment is caused by a random effect of fixed size thus not a function of the treatment effect size.

For the secondary simulation models (4) and (5) for both constant and learning treatment effects, we have obtained very similar results. The detailed results are presented in Supplementary Tables 1-4.

### Demonstrated application of the balancing procedure

We applied our 4-step strategy to balance site-level characteristics to the CARIBOU-2 study which tests the effectiveness of an ICP using the SWCRT with six sites as a demonstration. Step 1. We identified two potential site-level effect moderators, rurality and community income level. For rurality, there were two sites categorized as rural or semi-rural (level 1), two sites as rural and urban mixed (level 2), and two sites as urban or semi-urban (level 3). For income level, three sites were categorized as from low income communities (level 1), and the remaining three, medium income (level 2). When assessing multi-collinearity, we determined that the two measures were only moderately correlated (Spearman’s *ρ* = 0.41). Step 2. We decided to balance linear trends and seasonal effects with a cycle of 4 timepoints. Step 3. We assigned equal weights to each site-level characteristic and further, equal weights on the linear and seasonal trends. Step 4. The overall imbalance indices ranged from ${i}_{0}$ = 0.060 to 0.687 among the 720 permuted randomization sequences. Eight sequences tied for the lowest imbalance score of ${i}_{0}$ = 0.060 and another eight tied for the second lowest imbalance score of ${i}_{0}$ = 0.135. We randomly selected one of these sequences for the CARIBOU-2 trial. This maintained a reasonable level of uncertainty in the randomization process.

## Discussion

Our results demonstrate that pre-balancing site-level covariates in SWCRTs can increase efficiency; the efficiency loss is more pronounced when there is a learning effect vs. a constant effect in treatment efficacy. With the learning effect in place, the impact of sequential imbalance is amplified due to the confounding between site level characteristics and time. This magnitude of the bias is diminished when the effect size, sample size, or number of study sites increase. All scenarios showed no bias. Sensitivity analyses demonstrated that these trends hold for alternative models including random effects on the treatment effect at the time, and time-by-cluster levels. However, not all of the estimation bias can be associated with covariate imbalance alone as pointed by Kenny et al. [26].

We also proposed a unified framework to assess sequential, non-linear, and seasonal imbalances in site-level covariates. Our proposed imbalance indices are based on the (partial) correlation of the site-level covariate with sequential, quadratic, or cyclical time of crossover. One advantage is that our proposed imbalance indices all range from 0 (perfectly balanced) to 1 (perfectly imbalanced). In comparison, the imbalance metrics proposed by Lew et al. (2019) do not have a fixed range. Our strategy for determining the overall imbalance index across multiple site-level covariates and imbalance types requires guidance from the study team to determine the covariates of interest, time-trends to balance, and the relative weight of these parameters. This strategy mirrors the approach by Lew et al.

Our study has a few limitations. Our simulations assumed that a single site would crossover to the active intervention per step. Larger SWCRTs may have multiple sites crossover at each timepoint. By having multiple sites crossover per timepoint may diminish the effect of site-level covariate imbalances on estimation bias because the covariates would be averaged across multiple sites, thus reducing the random chance for imbalance. Our investigation focused on SWCRTs where the outcome is measured once per participant. We did not investigate an alternative design where participants would be followed longitudinally as they crossover from the control to the active intervention. Our study focuses on establishing and examining the pre-balancing study through simulations; we did not perform a formal comparison of pre-balancing versus covariate-constrained or stratified randomization. We have assumed only an ordinal scale for the hypothesized site characteristics with three levels (0, 1 and 2); this represents a highly varying effect size across sites. Smaller differences between sites or a continuous site characteristics may have different impacts on the findings.

Future research directions include examining the effect of: (1) non-linear and/or seasonal imbalances on estimation bias; (2) continuous or ordinal site-level characteristics with smaller differences between sites; (3) the bias in larger SWCRTs with multiple sites crossing over per timepoint; (4) covariate-constrained randomization versus pre-balancing; and (5) longitudinal SWCRT designs. For smaller SWCRTs, where the chance for random imbalance of site-level covariates is higher, we recommend investigators to carefully consider pre-balancing their covariates for sequential, non-linear, and/or seasonal effects to prevent efficiency loss in estimation.

## Conclusions

In summary, we have established a unified framework to quantify linear, non-linear, and seasonal imbalances of site-level characteristics in stepped-wedge cluster randomized trials. Our proposed balancing strategy will enable investigators to balance any combination of linear, non-linear, and seasonal trends. Our findings highlight the importance of pre-balancing site-level characteristics in order to minimize the potential for efficiency loss, especially for interventions with a learning effect.

### Supplementary Information

**Additional file 1: Table S1.** Relative Root Mean Square Error (RRMSE) of the estimated treatment effects. Simulated models include a random effect on treatment effect  at the site level. **Table S2.** Relative mean bias of the estimated treatment effects. Simulated models include a random effect on treatment effect at the site level. **Table S3.** Relative Root Mean Square Error (RRMSE) of the estimated treatment effects. Simulated models include a random intercept at the site by time level. **Table S4.** Relative mean bias of the estimated treatment effects. Simulated models include a random intercept at the site by time level.

**Additional file 2.** Code for calculating the imbalance scores.

## Data Availability

The datasets used and/or analysed during the current study are available from the corresponding author on reasonable request.

## Abbreviations

SWCRTs: Stepped-wedge cluster randomized trials
RRMSE: Relative root mean square error
CRTs: Cluster randomized trials
HIV: Human immunodeficiency virus
CIHR: Canadian Institutes of Health Research
ICP: Integrated care pathway
TAU: Treatment-as-usual
ICC: Intra-cluster correlation coefficient
CARIBOU-2: Effectiveness of an Integrated Care Pathway for Adolescent Depression: a Multi-site Stepped-Wedge, Cluster-Randomized Controlled Trial
