# Supplementary material for: Comparing analytical strategies for balancing site-level characteristics in stepped-wedge cluster randomized trials: a simulation study
Source: BMC Med Res Methodol. 2023 Sep 12;23:206. doi: 10.1186/s12874-023-02027-y (PMC10496299; doi:10.1186/s12874-023-02027-y)
Supplement: Supplementary file 1 — Additional file 1: Table S1. Relative Root Mean Square Error (RRMSE) of the estimated treatment effects. Simulated models include a random effect on treatment effect at the site level. Table S2. Relative mean bias of the estimated treatment effects. Simulated models include a random effect on treatment effect at the site level. Table S3. Relative Root Mean Square Error (RRMSE) of the estimated treatment effects. Simulated models include a random intercept at the site by time level. Table S4. Relative mean bias of the estimated treatment effects. Simulated models include a random intercept at the site by time level. [file 12874_2023_2027_MOESM1_ESM.docx]

Table S1: Relative Root Mean Square Error (RRMSE) of the estimated treatment effects. Simulated models include a random effect on treatment effect $\beta_{1}$ at the site level.

| ICC | Number of sites | Sample size at each step | Imbalance Index | Percentiles | With Learning Effect | | | With Constant Effect | | |
| --- | --- | --- | --- | --- | --- | --- | --- | --- | --- | --- |
|  |  |  |  |  | Effect Size | | | Effect Size | | |
|  |  |  |  |  | 0.2 | 0.5 | 1.0 | 0.2 | 0.5 | 1.0 |
| 0.01 | 6 | 10 | 0.000 | 0^th^ | 1.629 | 0.736 | 0.447 | 0.745 | 0.311 | 0.166 |
|  |  |  | 0.239 | 33^rd^ | 1.654 | 0.757 | 0.466 | 0.755 | 0.316 | 0.167 |
|  |  |  | 0.478 | 67^th^ | 1.742 | 0.858 | 0.550 | 0.778 | 0.328 | 0.172 |
|  |  |  | 0.717 | 83^rd^ | 1.820 | 1.013 | 0.692 | 0.804 | 0.343 | 0.176 |
|  |  |  | 0.956 | 100^th^ | 1.946 | 1.237 | 0.966 | 0.852 | 0.372 | 0.185 |
|  |  | 20 | 0.000 | 0^th^ | 1.299 | 0.604 | 0.380 | 0.560 | 0.234 | 0.127 |
|  |  |  | 0.239 | 33^rd^ | 1.325 | 0.636 | 0.402 | 0.565 | 0.236 | 0.128 |
|  |  |  | 0.478 | 67^th^ | 1.402 | 0.716 | 0.461 | 0.571 | 0.238 | 0.128 |
|  |  |  | 0.717 | 83^rd^ | 1.495 | 0.872 | 0.574 | 0.599 | 0.248 | 0.131 |
|  |  |  | 0.956 | 100^th^ | 1.655 | 1.118 | 0.803 | 0.635 | 0.260 | 0.135 |
|  | 12 | 10 | 0.000 | 0^th^ | 0.892 | 0.409 | 0.256 | 0.391 | 0.165 | 0.090 |
|  |  |  | 0.148 | 33^rd^ | 0.920 | 0.434 | 0.271 | 0.393 | 0.166 | 0.090 |
|  |  |  | 0.296 | 67^th^ | 0.958 | 0.484 | 0.312 | 0.402 | 0.170 | 0.092 |
|  |  |  | 0.414 | 83^rd^ | 0.999 | 0.546 | 0.362 | 0.407 | 0.171 | 0.091 |
|  |  |  | 0.946 | 100^th^ | 1.356 | 1.051 | 0.820 | 0.497 | 0.201 | 0.101 |
|  |  | 20 | 0.000 | 0^th^ | 0.715 | 0.340 | 0.220 | 0.296 | 0.126 | 0.071 |
|  |  |  | 0.148 | 33^rd^ | 0.740 | 0.359 | 0.231 | 0.296 | 0.126 | 0.071 |
|  |  |  | 0.296 | 67^th^ | 0.764 | 0.399 | 0.263 | 0.299 | 0.127 | 0.072 |
|  |  |  | 0.414 | 83^rd^ | 0.829 | 0.459 | 0.301 | 0.303 | 0.128 | 0.072 |
|  |  |  | 0.946 | 100^th^ | 1.227 | 0.953 | 0.656 | 0.340 | 0.137 | 0.075 |
| 0.10 | 6 | 10 | 0.000 | 0^th^ | 2.213 | 0.950 | 0.549 | 0.851 | 0.347 | 0.179 |
|  |  |  | 0.239 | 33^rd^ | 2.232 | 0.969 | 0.566 | 0.851 | 0.348 | 0.181 |
|  |  |  | 0.478 | 67^th^ | 2.317 | 1.055 | 0.644 | 0.860 | 0.352 | 0.182 |
|  |  |  | 0.717 | 83^rd^ | 2.364 | 1.170 | 0.770 | 0.858 | 0.351 | 0.182 |
|  |  |  | 0.956 | 100^th^ | 2.447 | 1.341 | 1.001 | 0.862 | 0.356 | 0.184 |
|  |  | 20 | 0.000 | 0^th^ | 1.675 | 0.742 | 0.442 | 0.620 | 0.255 | 0.135 |
|  |  |  | 0.239 | 33^rd^ | 1.692 | 0.766 | 0.461 | 0.618 | 0.255 | 0.136 |
|  |  |  | 0.478 | 67^th^ | 1.763 | 0.845 | 0.522 | 0.614 | 0.252 | 0.135 |
|  |  |  | 0.717 | 83^rd^ | 1.813 | 0.965 | 0.625 | 0.619 | 0.254 | 0.135 |
|  |  |  | 0.956 | 100^th^ | 1.933 | 1.162 | 0.828 | 0.624 | 0.256 | 0.136 |
|  | 12 | 10 | 0.000 | 0^th^ | 1.132 | 0.496 | 0.294 | 0.441 | 0.182 | 0.097 |
|  |  |  | 0.148 | 33^rd^ | 1.150 | 0.517 | 0.308 | 0.441 | 0.182 | 0.097 |
|  |  |  | 0.296 | 67^th^ | 1.193 | 0.566 | 0.350 | 0.444 | 0.184 | 0.098 |
|  |  |  | 0.414 | 83^rd^ | 1.208 | 0.612 | 0.395 | 0.439 | 0.181 | 0.096 |
|  |  |  | 0.946 | 100^th^ | 1.498 | 1.063 | 0.838 | 0.448 | 0.187 | 0.100 |
|  |  | 20 | 0.000 | 0^th^ | 0.838 | 0.383 | 0.239 | 0.322 | 0.135 | 0.075 |
|  |  |  | 0.148 | 33^rd^ | 0.863 | 0.402 | 0.250 | 0.321 | 0.134 | 0.075 |
|  |  |  | 0.296 | 67^th^ | 0.879 | 0.439 | 0.280 | 0.320 | 0.135 | 0.075 |
|  |  |  | 0.414 | 83^rd^ | 0.934 | 0.495 | 0.318 | 0.319 | 0.134 | 0.075 |
|  |  |  | 0.946 | 100^th^ | 1.275 | 0.959 | 0.670 | 0.318 | 0.135 | 0.076 |

Table S2: Relative mean bias of the estimated treatment effects. Simulated models include a random effect on treatment effect $\beta_{1}$ at the site level.

| ICC | Number of sites | Sample size at each step | Imbalance Index | Percentiles | With Learning Effect | | | With Constant Effect | | |
| --- | --- | --- | --- | --- | --- | --- | --- | --- | --- | --- |
|  |  |  |  |  | Effect Size | | | Effect Size | | |
|  |  |  |  |  | 0.2 | 0.5 | 1.0 | 0.2 | 0.5 | 1.0 |
| 0.01 | 6 | 10 | 0.000 | 0^th^ | 0.006 | 0.002 | 0.000 | 0.004 | 0.002 | 0.001 |
|  |  |  | 0.239 | 33^rd^ | 0.013 | 0.007 | 0.006 | 0.002 | 0.000 | 0.000 |
|  |  |  | 0.478 | 67^th^ | 0.009 | 0.004 | 0.000 | 0.003 | 0.000 | 0.000 |
|  |  |  | 0.717 | 83^rd^ | 0.006 | 0.001 | 0.002 | 0.003 | 0.001 | 0.001 |
|  |  |  | 0.956 | 100^th^ | 0.000 | 0.000 | 0.004 | 0.004 | 0.004 | 0.002 |
|  |  | 20 | 0.000 | 0^th^ | 0.004 | 0.001 | 0.003 | 0.002 | 0.001 | 0.001 |
|  |  |  | 0.239 | 33^rd^ | 0.036 | 0.018 | 0.007 | 0.003 | 0.000 | 0.000 |
|  |  |  | 0.478 | 67^th^ | 0.015 | 0.006 | 0.001 | 0.002 | 0.001 | 0.000 |
|  |  |  | 0.717 | 83^rd^ | 0.018 | 0.014 | 0.011 | 0.008 | 0.004 | 0.002 |
|  |  |  | 0.956 | 100^th^ | 0.001 | 0.002 | 0.002 | 0.002 | 0.000 | 0.000 |
|  | 12 | 10 | 0.000 | 0^th^ | 0.011 | 0.003 | 0.001 | 0.002 | 0.001 | 0.000 |
|  |  |  | 0.148 | 33^rd^ | 0.008 | 0.004 | 0.001 | 0.002 | 0.001 | 0.001 |
|  |  |  | 0.296 | 67^th^ | 0.009 | 0.003 | 0.001 | 0.006 | 0.002 | 0.001 |
|  |  |  | 0.414 | 83^rd^ | 0.008 | 0.006 | 0.004 | 0.001 | 0.000 | 0.000 |
|  |  |  | 0.946 | 100^th^ | 0.003 | 0.005 | 0.010 | 0.003 | 0.000 | 0.000 |
|  |  | 20 | 0.000 | 0^th^ | 0.004 | 0.002 | 0.001 | 0.002 | 0.001 | 0.000 |
|  |  |  | 0.148 | 33^rd^ | 0.007 | 0.002 | 0.002 | 0.001 | 0.000 | 0.000 |
|  |  |  | 0.296 | 67^th^ | 0.002 | 0.000 | 0.000 | 0.000 | 0.000 | 0.000 |
|  |  |  | 0.414 | 83^rd^ | 0.004 | 0.002 | 0.001 | 0.000 | 0.000 | 0.000 |
|  |  |  | 0.946 | 100^th^ | 0.007 | 0.000 | 0.001 | 0.001 | 0.001 | 0.000 |
| 0.10 | 6 | 10 | 0.000 | 0^th^ | 0.010 | 0.004 | 0.002 | 0.008 | 0.003 | 0.001 |
|  |  |  | 0.239 | 33^rd^ | 0.027 | 0.015 | 0.008 | 0.001 | 0.000 | 0.000 |
|  |  |  | 0.478 | 67^th^ | 0.006 | 0.002 | 0.000 | 0.006 | 0.001 | 0.000 |
|  |  |  | 0.717 | 83^rd^ | 0.012 | 0.002 | 0.001 | 0.005 | 0.002 | 0.001 |
|  |  |  | 0.956 | 100^th^ | 0.027 | 0.007 | 0.001 | 0.005 | 0.004 | 0.002 |
|  |  | 20 | 0.000 | 0^th^ | 0.004 | 0.001 | 0.004 | 0.001 | 0.001 | 0.001 |
|  |  |  | 0.239 | 33^rd^ | 0.059 | 0.027 | 0.012 | 0.003 | 0.001 | 0.000 |
|  |  |  | 0.478 | 67^th^ | 0.020 | 0.007 | 0.001 | 0.004 | 0.001 | 0.000 |
|  |  |  | 0.717 | 83^rd^ | 0.013 | 0.011 | 0.010 | 0.008 | 0.003 | 0.002 |
|  |  |  | 0.956 | 100^th^ | 0.004 | 0.002 | 0.001 | 0.002 | 0.001 | 0.000 |
|  | 12 | 10 | 0.000 | 0^th^ | 0.006 | 0.001 | 0.000 | 0.001 | 0.000 | 0.000 |
|  |  |  | 0.148 | 33^rd^ | 0.016 | 0.008 | 0.003 | 0.005 | 0.002 | 0.001 |
|  |  |  | 0.296 | 67^th^ | 0.006 | 0.000 | 0.001 | 0.005 | 0.002 | 0.001 |
|  |  |  | 0.414 | 83^rd^ | 0.007 | 0.006 | 0.004 | 0.003 | 0.001 | 0.001 |
|  |  |  | 0.946 | 100^th^ | 0.008 | 0.001 | 0.006 | 0.002 | 0.000 | 0.000 |
|  |  | 20 | 0.000 | 0^th^ | 0.002 | 0.002 | 0.001 | 0.002 | 0.001 | 0.000 |
|  |  |  | 0.148 | 33^rd^ | 0.007 | 0.002 | 0.002 | 0.001 | 0.001 | 0.000 |
|  |  |  | 0.296 | 67^th^ | 0.002 | 0.001 | 0.001 | 0.000 | 0.000 | 0.000 |
|  |  |  | 0.414 | 83^rd^ | 0.008 | 0.003 | 0.002 | 0.001 | 0.001 | 0.000 |
|  |  |  | 0.946 | 100^th^ | 0.011 | 0.003 | 0.001 | 0.002 | 0.001 | 0.000 |

Table S3: Relative Root Mean Square Error (RRMSE) of the estimated treatment effects. Simulated models include a random intercept at the site by time level.

| ICC | Number of sites | Sample size at each step | Imbalance Index | Percentiles | With Learning Effect | | | With Constant Effect | | |
| --- | --- | --- | --- | --- | --- | --- | --- | --- | --- | --- |
|  |  |  |  |  | Effect Size | | | Effect Size | | |
|  |  |  |  |  | 0.2 | 0.5 | 1.0 | 0.2 | 0.5 | 1.0 |
| 0.01 | 6 | 10 | 0.000 | 0^th^ | 1.610 | 0.725 | 0.459 | 0.781 | 0.328 | 0.184 |
|  |  |  | 0.239 | 33^rd^ | 1.600 | 0.747 | 0.507 | 0.779 | 0.331 | 0.182 |
|  |  |  | 0.478 | 67^th^ | 1.698 | 0.916 | 0.728 | 0.808 | 0.358 | 0.199 |
|  |  |  | 0.717 | 83^rd^ | 1.844 | 1.130 | 0.981 | 0.859 | 0.410 | 0.233 |
|  |  |  | 0.956 | 100^th^ | 1.960 | 1.348 | 1.238 | 0.904 | 0.480 | 0.266 |
|  |  | 20 | 0.000 | 0^th^ | 1.296 | 0.602 | 0.398 | 0.595 | 0.253 | 0.149 |
|  |  |  | 0.239 | 33^rd^ | 1.326 | 0.645 | 0.460 | 0.614 | 0.261 | 0.148 |
|  |  |  | 0.478 | 67^th^ | 1.423 | 0.824 | 0.688 | 0.630 | 0.277 | 0.161 |
|  |  |  | 0.717 | 83^rd^ | 1.550 | 1.043 | 0.950 | 0.650 | 0.307 | 0.183 |
|  |  |  | 0.956 | 100^th^ | 1.741 | 1.303 | 1.227 | 0.717 | 0.368 | 0.206 |
|  | 12 | 10 | 0.000 | 0^th^ | 0.884 | 0.406 | 0.262 | 0.402 | 0.178 | 0.114 |
|  |  |  | 0.148 | 33^rd^ | 0.913 | 0.443 | 0.312 | 0.405 | 0.180 | 0.115 |
|  |  |  | 0.296 | 67^th^ | 0.958 | 0.536 | 0.433 | 0.417 | 0.185 | 0.115 |
|  |  |  | 0.414 | 83^rd^ | 1.019 | 0.633 | 0.546 | 0.435 | 0.196 | 0.119 |
|  |  |  | 0.946 | 100^th^ | 1.432 | 1.182 | 1.138 | 0.548 | 0.269 | 0.132 |
|  |  | 20 | 0.000 | 0^th^ | 0.725 | 0.343 | 0.231 | 0.311 | 0.145 | 0.101 |
|  |  |  | 0.148 | 33^rd^ | 0.746 | 0.381 | 0.283 | 0.318 | 0.146 | 0.099 |
|  |  |  | 0.296 | 67^th^ | 0.800 | 0.483 | 0.410 | 0.321 | 0.149 | 0.102 |
|  |  |  | 0.414 | 83^rd^ | 0.877 | 0.594 | 0.533 | 0.336 | 0.154 | 0.102 |
|  |  |  | 0.946 | 100^th^ | 1.312 | 1.151 | 1.124 | 0.409 | 0.189 | 0.099 |
| 0.10 | 6 | 10 | 0.000 | 0^th^ | 2.148 | 0.893 | 0.507 | 0.894 | 0.365 | 0.196 |
|  |  |  | 0.239 | 33^rd^ | 2.123 | 0.907 | 0.550 | 0.884 | 0.361 | 0.193 |
|  |  |  | 0.478 | 67^th^ | 2.221 | 1.055 | 0.757 | 0.897 | 0.369 | 0.202 |
|  |  |  | 0.717 | 83^rd^ | 2.335 | 1.250 | 1.003 | 0.909 | 0.382 | 0.220 |
|  |  |  | 0.956 | 100^th^ | 2.415 | 1.449 | 1.255 | 0.899 | 0.387 | 0.229 |
|  |  | 20 | 0.000 | 0^th^ | 1.638 | 0.696 | 0.421 | 0.668 | 0.282 | 0.161 |
|  |  |  | 0.239 | 33^rd^ | 1.670 | 0.739 | 0.481 | 0.681 | 0.283 | 0.158 |
|  |  |  | 0.478 | 67^th^ | 1.740 | 0.891 | 0.698 | 0.672 | 0.282 | 0.163 |
|  |  |  | 0.717 | 83^rd^ | 1.841 | 1.097 | 0.951 | 0.661 | 0.280 | 0.170 |
|  |  |  | 0.956 | 100^th^ | 2.019 | 1.351 | 1.227 | 0.673 | 0.288 | 0.178 |
|  | 12 | 10 | 0.000 | 0^th^ | 1.118 | 0.475 | 0.277 | 0.459 | 0.196 | 0.119 |
|  |  |  | 0.148 | 33^rd^ | 1.145 | 0.508 | 0.324 | 0.457 | 0.197 | 0.120 |
|  |  |  | 0.296 | 67^th^ | 1.180 | 0.586 | 0.437 | 0.457 | 0.195 | 0.118 |
|  |  |  | 0.414 | 83^rd^ | 1.226 | 0.670 | 0.544 | 0.466 | 0.199 | 0.120 |
|  |  |  | 0.946 | 100^th^ | 1.578 | 1.190 | 1.122 | 0.463 | 0.199 | 0.113 |
|  |  | 20 | 0.000 | 0^th^ | 0.865 | 0.379 | 0.235 | 0.346 | 0.157 | 0.105 |
|  |  |  | 0.148 | 33^rd^ | 0.876 | 0.410 | 0.285 | 0.349 | 0.157 | 0.103 |
|  |  |  | 0.296 | 67^th^ | 0.918 | 0.501 | 0.407 | 0.346 | 0.157 | 0.104 |
|  |  |  | 0.414 | 83^rd^ | 0.983 | 0.603 | 0.526 | 0.351 | 0.157 | 0.102 |
|  |  |  | 0.946 | 100^th^ | 1.374 | 1.143 | 1.106 | 0.339 | 0.144 | 0.085 |

Table S4: Relative mean bias of the estimated treatment effects. Simulated models include a random intercept at the site by time level.

| ICC | Number of sites | Sample size at each step | Imbalance Index | Percentiles | With Learning Effect | | | With Constant Effect | | |
| --- | --- | --- | --- | --- | --- | --- | --- | --- | --- | --- |
|  |  |  |  |  | Effect Size | | | Effect Size | | |
|  |  |  |  |  | 0.2 | 0.5 | 1.0 | 0.2 | 0.5 | 1.0 |
| 0.01 | 6 | 10 | 0.000 | 0^th^ | 0.008 | 0.003 | 0.001 | 0.007 | 0.004 | 0.002 |
|  |  |  | 0.239 | 33^rd^ | 0.006 | 0.006 | 0.005 | 0.000 | 0.001 | 0.001 |
|  |  |  | 0.478 | 67^th^ | 0.005 | 0.002 | 0.005 | 0.008 | 0.002 | 0.001 |
|  |  |  | 0.717 | 83^rd^ | 0.021 | 0.007 | 0.002 | 0.000 | 0.001 | 0.001 |
|  |  |  | 0.956 | 100^th^ | 0.018 | 0.017 | 0.017 | 0.010 | 0.005 | 0.003 |
|  |  | 20 | 0.000 | 0^th^ | 0.011 | 0.007 | 0.005 | 0.014 | 0.005 | 0.002 |
|  |  |  | 0.239 | 33^rd^ | 0.011 | 0.003 | 0.001 | 0.004 | 0.002 | 0.000 |
|  |  |  | 0.478 | 67^th^ | 0.005 | 0.004 | 0.004 | 0.009 | 0.004 | 0.002 |
|  |  |  | 0.717 | 83^rd^ | 0.019 | 0.016 | 0.015 | 0.007 | 0.004 | 0.002 |
|  |  |  | 0.956 | 100^th^ | 0.021 | 0.010 | 0.006 | 0.012 | 0.003 | 0.002 |
|  | 12 | 10 | 0.000 | 0^th^ | 0.001 | 0.001 | 0.000 | 0.006 | 0.003 | 0.001 |
|  |  |  | 0.148 | 33^rd^ | 0.002 | 0.003 | 0.003 | 0.003 | 0.001 | 0.000 |
|  |  |  | 0.296 | 67^th^ | 0.009 | 0.002 | 0.001 | 0.002 | 0.001 | 0.001 |
|  |  |  | 0.414 | 83^rd^ | 0.020 | 0.010 | 0.007 | 0.000 | 0.001 | 0.001 |
|  |  |  | 0.946 | 100^th^ | 0.006 | 0.007 | 0.008 | 0.013 | 0.006 | 0.003 |
|  |  | 20 | 0.000 | 0^th^ | 0.004 | 0.001 | 0.000 | 0.002 | 0.001 | 0.001 |
|  |  |  | 0.148 | 33^rd^ | 0.001 | 0.001 | 0.002 | 0.001 | 0.000 | 0.001 |
|  |  |  | 0.296 | 67^th^ | 0.005 | 0.005 | 0.005 | 0.007 | 0.003 | 0.002 |
|  |  |  | 0.414 | 83^rd^ | 0.010 | 0.003 | 0.001 | 0.003 | 0.001 | 0.000 |
|  |  |  | 0.946 | 100^th^ | 0.003 | 0.001 | 0.001 | 0.003 | 0.001 | 0.001 |
| 0.10 | 6 | 10 | 0.000 | 0^th^ | 0.016 | 0.005 | 0.002 | 0.009 | 0.003 | 0.001 |
|  |  |  | 0.239 | 33^rd^ | 0.013 | 0.008 | 0.006 | 0.003 | 0.001 | 0.001 |
|  |  |  | 0.478 | 67^th^ | 0.004 | 0.005 | 0.005 | 0.006 | 0.002 | 0.000 |
|  |  |  | 0.717 | 83^rd^ | 0.030 | 0.008 | 0.002 | 0.003 | 0.001 | 0.001 |
|  |  |  | 0.956 | 100^th^ | 0.005 | 0.012 | 0.015 | 0.004 | 0.003 | 0.003 |
|  |  | 20 | 0.000 | 0^th^ | 0.008 | 0.005 | 0.004 | 0.011 | 0.004 | 0.001 |
|  |  |  | 0.239 | 33^rd^ | 0.020 | 0.008 | 0.004 | 0.003 | 0.001 | 0.000 |
|  |  |  | 0.478 | 67^th^ | 0.007 | 0.001 | 0.004 | 0.009 | 0.003 | 0.002 |
|  |  |  | 0.717 | 83^rd^ | 0.018 | 0.015 | 0.014 | 0.001 | 0.002 | 0.002 |
|  |  |  | 0.956 | 100^th^ | 0.016 | 0.007 | 0.005 | 0.012 | 0.005 | 0.002 |
|  | 12 | 10 | 0.000 | 0^th^ | 0.003 | 0.001 | 0.000 | 0.007 | 0.003 | 0.002 |
|  |  |  | 0.148 | 33^rd^ | 0.009 | 0.005 | 0.004 | 0.002 | 0.001 | 0.000 |
|  |  |  | 0.296 | 67^th^ | 0.024 | 0.008 | 0.003 | 0.001 | 0.001 | 0.001 |
|  |  |  | 0.414 | 83^rd^ | 0.023 | 0.011 | 0.007 | 0.001 | 0.001 | 0.001 |
|  |  |  | 0.946 | 100^th^ | 0.009 | 0.008 | 0.008 | 0.013 | 0.005 | 0.003 |
|  |  | 20 | 0.000 | 0^th^ | 0.008 | 0.003 | 0.001 | 0.003 | 0.001 | 0.001 |
|  |  |  | 0.148 | 33^rd^ | 0.006 | 0.001 | 0.001 | 0.001 | 0.000 | 0.001 |
|  |  |  | 0.296 | 67^th^ | 0.001 | 0.004 | 0.005 | 0.005 | 0.003 | 0.002 |
|  |  |  | 0.414 | 83^rd^ | 0.011 | 0.004 | 0.002 | 0.003 | 0.001 | 0.000 |
|  |  |  | 0.946 | 100^th^ | 0.005 | 0.002 | 0.001 | 0.002 | 0.001 | 0.001 |
