# Supplementary material for: Comparing analytical strategies for balancing site-level characteristics in stepped-wedge cluster randomized trials: a simulation study
Source: BMC Med Res Methodol. 2023 Sep 12;23:206. doi: 10.1186/s12874-023-02027-y (PMC10496299; doi:10.1186/s12874-023-02027-y)
Supplement: Supplementary file 2 — Additional file 2. Code for calculating the imbalance scores. [file 12874_2023_2027_MOESM2_ESM.docx]

**Code for calculating the imbalance scores**

The following codes define three R functions, i.L ( ), i.Q ( ) and i.S ( ), which can be used to calculate the linear/sequential, quadratic, and seasonal imbalance indices respectively. The examples demonstrated in Figure 2 are used as examples at the end.

1. **A function for imbalance index for linear/sequential effect**

# Z indicates the site-level characteristics

i.L <- function ( Z ) {

length <- length ( Z )

tl <- seq ( length )

return ( abs(cor(rank ( Z ), tl) ) )

}

1. **A function for imbalance index for quadratic effect with linear effect controlled.**

# Z indicates the site-level characteristics

i.Q <- function ( Z ) {

partial <- function(X,Y,Z){

num <- abs ( cor(X,Y) - (cor(X,Z)*cor(Y,Z)) )

den <- sqrt((1 - cor(X,Z)^2)*(1 - cor(Y,Z)^2))

return ( abs(num/den) )

}

length <- length ( Z )

tl <- seq ( length )

tq <- tl^2

return ( partial ( rank( Z ), tq, tl ) )

}

1. **A function for imbalance index for seasonal effect with linear effect controlled.**

# Z indicates the site-level characteristics

# You may enter the season length. The default value is 4.

i.S <- function ( Z, season = 4 ) {

partial <- function(X,Y,Z){

num <- abs ( cor(X,Y) - (cor(X,Z)*cor(Y,Z)) )

den <- sqrt((1 - cor(X,Z)^2)*(1 - cor(Y,Z)^2))

return ( abs(num/den) )

}

length <- length ( Z )

tl <- seq ( length )

ts <- rep ( c (1:season), length/season + 1 ) [ 1:length]

return ( partial ( rank( Z ), ts, tl ) )

}

**# Examples are based on those sequences using defined functions in Figure 2**

# 2.a

Z <- c ( 0, 0, 0, 1, 0, 1, 1, 1, 2, 2, 2, 2)

i.L ( Z ) # 0.917

i.Q ( Z ) # 0.097

i.S ( Z ) # 0.061

# 2.b

Z <- c ( 2, 2, 1, 1, 0, 0, 0, 0, 1, 1, 2, 2)

i.L ( Z ) # 0.000

i.Q ( Z ) # 0.929

i.S ( Z ) # 0.000

# 2.c

Z <- c ( 0, 1, 2, 2, 0, 1, 1, 2, 0, 0, 1, 2)

i.L ( Z ) # 0.059

i.Q ( Z ) # 0.000

i.S ( Z ) # 0.946

# 2.d

Z <- c ( 1, 2, 0, 1, 0, 2, 0, 2, 1, 2, 0, 1)

i.L ( Z ) # 0.000

i.Q ( Z ) # 0.000

i.S ( Z ) # 0.000
